# Capt. Victor Moreau Rice

**Published:** 1918-12

**Authors:** 


**Capt. Victor Moreau Rice,** Buffalo 1904, of Batavia, died of pneumonia, complicating influenza, at the General Hospital at Ft. Oglethorpe, Oct. 13, aged 38. He was taken sick while a student officer at Camp Greenleaf.

